# Hawthorne Effect in Screening Ambulatory Activity Status Using Wearable Pedometers Varies Across Age Groups

**DOI:** 10.1016/j.focus.2026.100496

**Published:** 2026-03-23

**Authors:** Saleh Hammad, Abdelhafez Alnawayseh, Rami Hammad, Laurent Ballaz, Haidar Djemai

**Affiliations:** 1 Department of Physical and Health Education, Faculty of Educational Sciences, Al-Ahliyya Amman University, Amman, Jordan; 2 Department of Administration and Curriculum, Program of Sports Management and Training, Faculty of Arts and Educational Sciences, Middle East University, Jordan; 3 Sports performance expertise and analysis laboratory (LEAPS), University of Abdelhamid Mehri Constantine 2, New Town Ali Mendjeli, Constantine, Algeria; 4 Department of Sport Rehabilitation, Faculty of Sport Science, Mutah University, Mutah, Jordan; 5 Department of Biology, Faculty of Science, University of Quebec at Montreal, Montreal, Quebec, Canada; 6 Department of Kinesiology, Faculty of Science, University of Quebec at Montreal, Montreal, Quebec, Canada; 7 Research Center of the Montreal University Geriatric Institute, Montreal, Quebec, Canada; 8 Institute for Research in BioMedicine and Epidemiology of Sport (IRMES), Paris, France; 9 HealthFex, Toxicology Pharmacology and Cell Signaling, University Paris Cité, Paris, France; 10 Laboratory of Biology and Molecular Genetics, Medical School, University Salah Boubenider Constantine 3, New Town Ali Mendjeli, Constantine, Algeria; 11 School of Sport Science, University of Jordan, Amman, Jordan

**Keywords:** Sedentary lifestyle, technology, physical assessment, behavioral change, health

## Abstract

**Introduction:**

Using wearable step-counting devices is a common method in scientific research to evaluate ambulatory activity. The *Hawthorne effect*, defined as an increase in productivity or performance resulting from the perception of being monitored, is often overlooked by researchers and remains poorly understood, particularly in the assessment of ambulatory activity using wearable step-counting devices. This study aimed to assess ambulatory activity levels in various age groups and quantify the impact of wearing step-counting devices on the daily ambulatory activity in men.

**Methods:**

A convenience sample of 60 men was included and allocated into 3 age groups (20–25, 40–45, and 60–65 years), with 10 participants in the control group and 10 in the experimental group for each age category. Step count data were collected in 2 stages: first, from the previous week, month, and 6 months; second, after 7 days of wearing a digital sham pedometer watch. Participants in the experimental groups were told that the watch is a step-counting device and that their activity was being monitored, even though all data were collected from the iPhone “Health” application (iOS 16.5), while the control group remained blinded until the last data collection was completed.

**Results:**

Comparison of pre- and postintervention data showed positive Hawthorne effect on the level of ambulatory activity in young, middle-aged, and older adults by 62.90%, 55.58%, and 81.96%, respectively. Significant differences were found between each experimental group and its respective control group after the 1-week intervention (*p*<0.001).

**Conclusions:**

The research concludes that all experimental groups increased their step count by more than 50% compared with baseline levels.

## HIGHLIGHTS

• Perceived monitoring via pedometer increases ambulatory activity in men.
• The Hawthorne effect varies across different age groups.
• Pedometer-based active or inactive classification may be inaccurate.

## Graphical abstract

**Figure:**
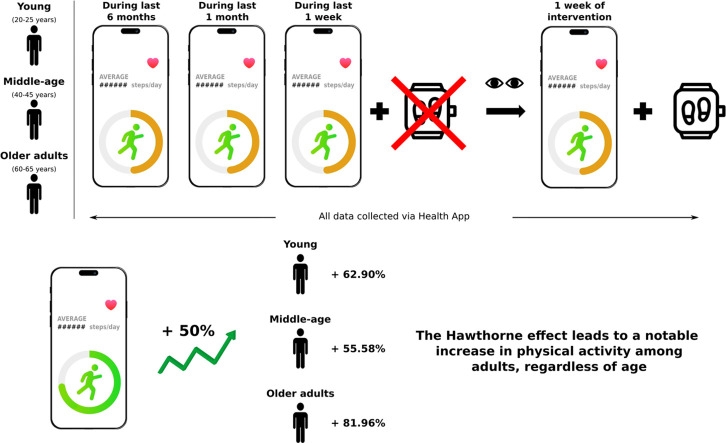

## INTRODUCTION

Regular physical activity significantly reduces the likelihood of developing chronic diseases such as diabetes, cardiovascular, and pulmonary diseases.^1^ The World Health Organization recommends that adults engage in approximately 150 minutes of moderate-intensity physical activity or 75 minutes of moderate-to-high-intensity physical activity each week.^2^ Among all physical activities, walking is one of the most recommended because it is an ideal activity for interventions targeting both intensity and frequency.^3^^,^^4^ A higher daily step count at a speed above 5 km/hour was significantly associated with lower mortality.^5^^,^^6^ Traditional recommendation of 10,000 walking steps per day is the minimum for an active person.^7^ A large-scale meta-analysis by Ding et al.^4^ demonstrated a clear and graded association between daily step count and health status, with substantial reductions in mortality, cardiovascular and cognitive disease risks from as low as 7,000 steps per day. Given these recommendations and the well-established health benefits of walking, it is essential to be able to measure step count to objectively assess ambulatory activity.

In clinical research, self-reported physical activity data are not recommended because of recall bias.^8^ Alternatively, pedometers, which use body-worn motion sensors to objectively measure step counts, such as the Fitbit tracker, offer a simple and inexpensive method to accurately assess ambulatory activity.^5^^,^^6^^,^^9^, ^10^, ^11^ Step counts are usually measured over a 1-week period to provide a meaningful evaluation of ambulatory activity.^12^^,^^13^ Today, most pedometers are integrated into a watch, whereas the gold standard measure is based on accelerometer measurements attached to the shoes.^12^^,^^14^ Recently, smartphone applications have gained in popularity because of technological advances and their convenience in measuring ambulatory activity.^8^^,^^9^

A potential confounding factor when using physical activity trackers is the Hawthorne Effect (HE), in which individuals’ awareness of being monitored can lead to increased productivity. This phenomenon was first identified in industrial settings, where the HE was defined as an increase in worker productivity stemming from psychological stimuli.^15^ The definition was later extended to include changes in behavior resulting from the awareness of being observed not only in the industrial settings but also in various fields.^16^ In the context of ambulatory monitoring, the HE suggests that a person may take more steps than usual simply because they know they are being monitored. A previous meta-analysis, focusing on pedometer measurements, showed that wearing a pedometer could result in a 2,000-step increase per day, depending on data collecting times.^17^ However, a research article showed an increase in maximum step count during a week of using a pedometer (median: 9,834 steps) compared with baseline (median: 6,963 steps) among middle-aged men and women; data were obtained from the pedometers by connecting it to the mobile application.^18^ This increase may produce inaccurate results in studies that categorize participants as active and inactive based on the results of step-counting devices. Previous studies have not quantified the exact proportion of total activity increase attributed to the use of pedometer watches, and differences among different age groups are still missing in literature.

Given the lack of information on the precise percentage of the HE when using pedometer watches in changing ambulatory activity performance in different age groups,^19^ this study aimed to evaluate the HE during the use of digital sham pedometer watches on ambulatory activity in adult men across different age groups.

## METHODS

### Study Sample

A convenience sample of 60 healthy adult men was included in the present study. Only men were included in the study given the significant differences in lifestyle between men and women, including differences in ambulatory activity.^20^ Participants belonged to 3 age groups (young: 20–25 years, middle age: 40–45 years, and older adults: 60–65 years) (Table 1). For each age group, participants were randomly allocated to an experimental group (Exp, *n*=10) and a control group (Ctrl, *n*=10). All participants previously assigned in the participant bank of the Department of Physical and Health Education received an email for participation with all the inclusion criteria. Participants were required to meet the following criteria to be included in the current study: (1) have an activated “Health” application on their iPhone, (2) be within the targeted age groups, (3) have performed fewer than 10,000 steps per day on average over the past 6 months,^21^, ^22^, ^23^ and (4) have no reported physical or mental disabilities or known illnesses. The Institutional Review Board (Faculty of Educational Sciences, Al-Ahliyya Amman University) approved this study according to protocol (No.: FES-18G-291). All participants provided their written informed consent before taking part in the study and to allow their anonymized data to be used for research and publication purposes. The study complied with the institutional committee’s ethical standards and followed the principles outlined in the Declaration of Helsinki.

**Table 1:** Characteristics of the Participants

Age, years	Groups	Height, cm	Weight, kg	BMI
20–25	Exp	175.30±5.74	71.50±8.32	23.21±1.79
	Ctrl	176.36±6.92	70.55±6.96	22.71±2.20
40–45	Exp	175.00±1.83	81.70±3.06	26.70±1.50
	Ctrl	173.30±1.89	81.00±3.37	27.00±1.68
60–65	Exp	169.90±1.79	82.80±2.62	28.71±1.45
	Ctrl	169.80±1.81	84.40±3.37	29.29±1.42

Ctrl, control; Exp, experimental.

### Measures

Step count data were collected at 4 time points over a 6-month period to ensure consistency in participants’ habitual activity levels: 6 months, 1 month, 1 week before the intervention, and immediately after the 1-week intervention. Data were extracted from the preinstalled Health app on participants’ iPhones (iOS 16.5), which has demonstrated high reliability for tracking daily steps and distance across different phone placements.^24^, ^25^, ^26^, ^27^ Participants were randomly assigned to experimental or control groups. One day before the intervention, all participants attended a baseline session at the Al-Ahliyya Amman University, held in separate locations by group, to provide initial data and sign consent forms. During the 7-day intervention, participants in the experimental group wore digital sham pedometer watches (eYotto digital sports watches, 0.96 inches, Model 1099) and were informed that their step counts would be recorded daily by the device. In contrast, control participants provided step counts data and scheduled the follow-up session without being explicitly informed about the monitoring to minimize awareness effects.

All measurements were conducted on the same day and at the same time for both groups. Participants returned 9 days after the baseline visit for postintervention data collection. To prevent potential overperformance bias, data from the 2 visit days were excluded, and only the average step count from the 7 full intervention days was included in the final analysis. During a brief interview with the study authors, participants stated that no unusual events occurred during the intervention week and that they did not have their phones with them during their sport activities.

### Statistical Analysis

All statistical analyses were conducted using R software (Version 4.4.1; R Foundation for Statistical Computing, Vienna, Austria). Data are presented as mean ± SD. A linear mixed-effects model (LMM) was used to examine the effects of time, Exp/Ctrl group, and age group, as well as their interactions, on participants’ daily step count. The model was fitted using the nlme package (lme function), with participant ID as a random intercept. Adjusted group and time comparisons were obtained using least-squares means from the mixed-effects model (glht function, *multcomp* package) with Bonferroni-adjusted post-hoc tests. In addition, an exploratory cross-sectional study was conducted within the experimental group to assess the relationship between age and the mean number of steps taken daily before and after the intervention. Pearson correlation coefficient was calculated, and a simple linear regression was performed using the mean number of daily steps at baseline (before the intervention) as the dependent variable and age (in years) as the predictor. This analysis was exploratory and is presented only for the experimental group for descriptive purposes. Statistical significance was set at *p*<0.05.

A post-hoc power analysis was conducted using the observed means (reference values) and SDs. For each age group, an estimate of the experimental mean was made by applying the observed percentage change (62.9%, 55.6%, and 82.0% for young adults, middle-aged adults, and older adults, respectively) to the 1-week reference value. Cohen *d* values ​​were then calculated using pooled SDs, assuming equal sample sizes (*n*=10 for each group). The resulting *d* values ​​were 2.93, 9.81, and 6.93 for young adults, middle-aged adults, and older adults, respectively. These 3 *d* values ​​correspond to post-hoc power estimates greater than 0.99, assuming a two-tailed independent samples *t*-test (α=0.05). These data indicate that the study had sufficient power to detect such large effects between the experimental and control groups, despite a relatively small sample size.

## RESULTS

Data from 60 healthy men were included in this study, with 10 participants assigned to the experimental group and 10 to the control group within each age group. The mean and SD of height, body weight, and BMI for each age group are presented in Table 1. No significant differences were observed between the experimental and control groups within each age group for all variables, and no significant differences in height were observed across groups.

The within-group before intervention/baseline data indicate no significant difference across the 3 different measurement points. Moreover, the baseline values were similar between the experimental and control groups, except at 1 month before the intervention of the younger group. However, it was different on the baseline data between older group and the young and middle age groups, with lower steps performed by the older group (Table 2).

**Table 2:** Means and SDs for the Step Count Variable Distributed According to the Research Groups in the 3 Measurements

Age	Group	Measurement time before		
		6 Months	1 Month	1 Week
**Young**	**Exp**	5,548.40±901.44	**5,093.80±937.68**	5,545.10±723.65
	**Ctrl**	5,038.20±1,821.14	4,743.80±1,893.03	4,853.90±1,880.08
**Middle age**	**Exp**	5,557.70±405.46	5,423.60±446.46	5,422.90±308.77
	**Ctrl**	5,535.20±354.38	5,466.80±262.38	5,510.10±287.75
**Older adults**	**Exp**	3,817.50±427.90	3,781.00±365.64	3,772.20±384.54
	**Ctrl**	3,762.70±503.67	3,842.20±474.92	3,782.80±497.75

*Note:* Values are presented as mean ± SD. Boldface indicates statistical significance (*p*<0.05); linear mixed models with repeated-measures ANOVA, post-hoc Bonferroni correction.

Ctrl, control; Exp, experimental.

**Figure 1:**
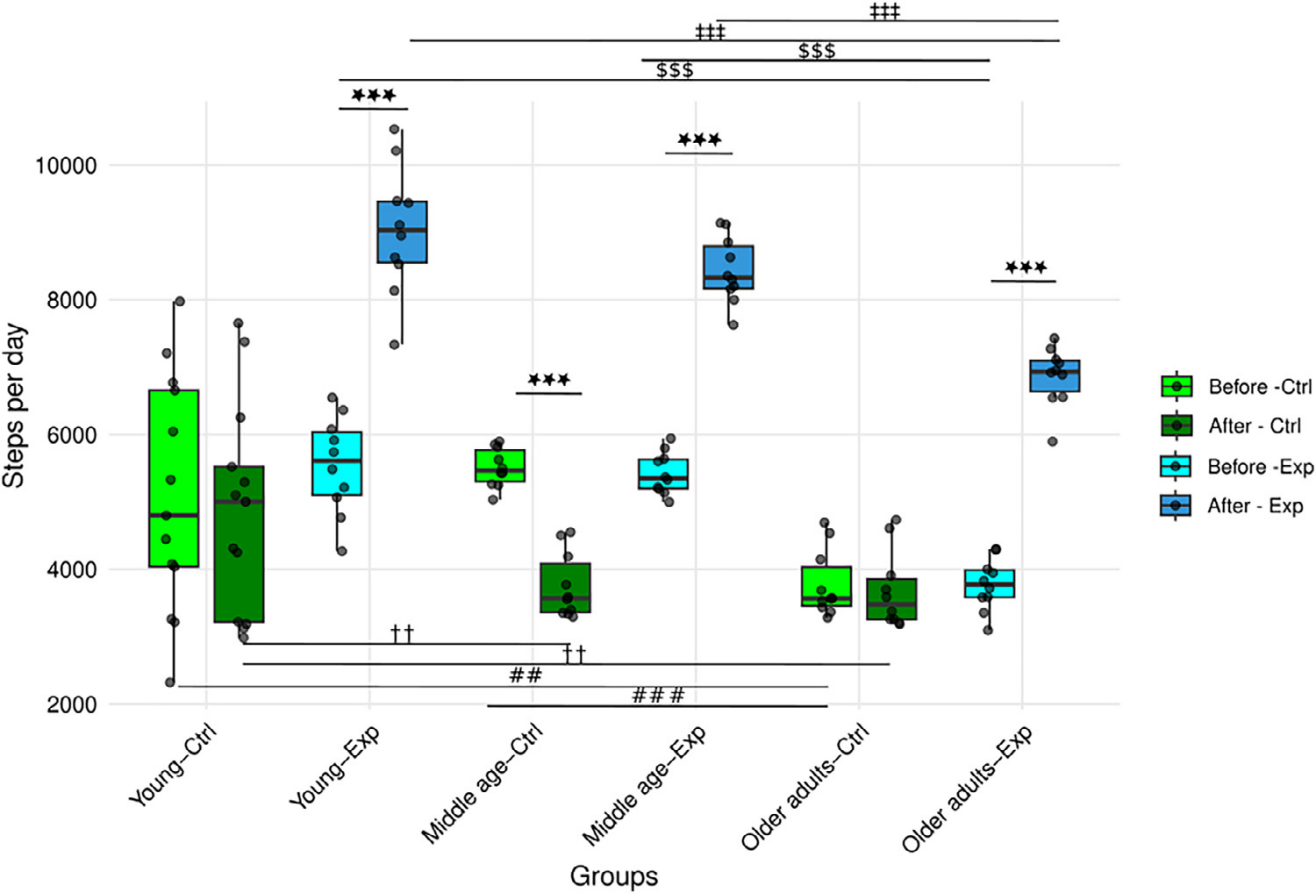
Number of steps taken daily for the experimental and control research groups before (1 week before) and after wearing the watch. *Note:* Each group (Exp in blue and Ctrl in green) was evaluated before and after 1 week of wearing the watch. Young: 20–25 y; Middle age: 40–45 y; Older adults: 60–65 y. Before versus After= ***p<0.001; Age - Exp - Before= $$$*p*<0.001; Age - Exp - After= ‡‡‡*p*<0.001; Age - Ctrl - Before= ##*p*<0.01, ###*p*<0.001; Age - Ctrl - After= †† (Linear mixed models with repeated-measures ANOVA, post-hoc Bonferroni correction). Ctrl, control; Exp, experimental.

After the 7-day intervention period, postintervention data were compared to baseline values collected 1 week before using a LMM with group (experimental versus control), time (pre versus post), and age group (young, middle-aged, older) as fixed factors. A significant group X time interaction was observed (*p*<0.05), indicating that wearing a pedometer watch increased daily step counts in men compared with baseline (Figure 1). The relative change (%) in daily steps was calculated as: (after − before) / before X 100).

Based on this calculation, significant increases (*p*<0.05) were observed across all experimental age groups, with differences between them. Specifically, young male adults increased their daily step counts by 62.9%, middle-aged adults by 55.6%, and older adults by 82.0%. However, after Bonferroni adjustment, the change in the middle-aged group was not statistically significant.

Fixed-effect estimates further indicated that participants in the experimental group increased their step count by an average of +477.7 steps/day (SE=39.4, *p*<0.001), whereas the control group remained relatively unchanged. Interaction analyses revealed that this effect varied by age group, with older adults showing a larger and significant performance-by-time interaction (β=+137.6, SE=56.3, *p*=0.015), suggesting that older participants were more behaviorally responsive to the awareness of being monitored. In contrast, postintervention data in the control groups remained stable, except for the middle-aged control group, which showed a slight but significant decrease in daily steps (Figure 1).

**Figure 2:**
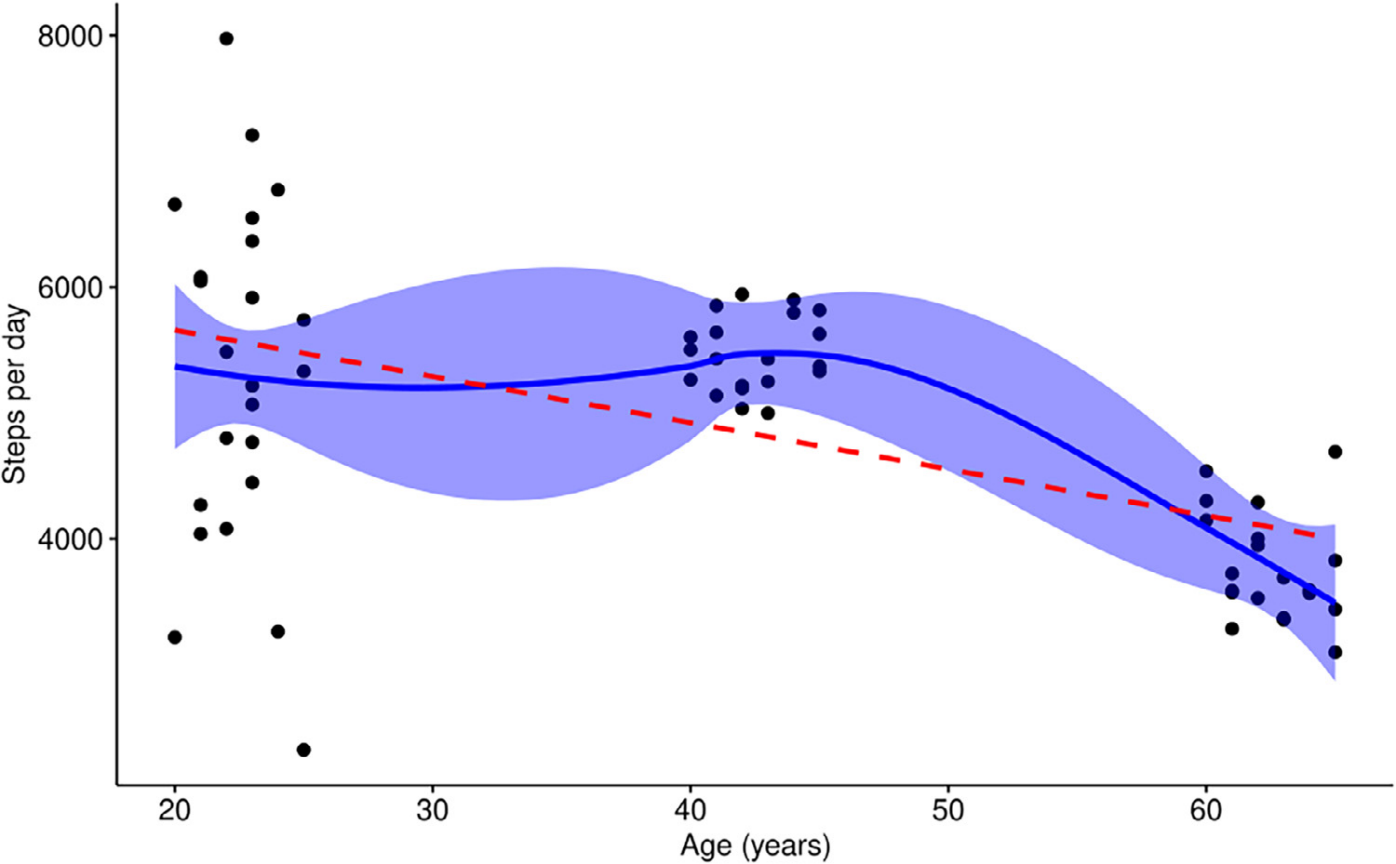
Association between age and number of steps performed daily for all groups, 1 week before wearing the watch. *Note:* The scatter plot illustrates the relationship between age and average daily step count. Data collected prior to wearing the watch are represented by blue circles. Linear regression lines (broken red line), with 95% CIs (shaded areas) are shown for each time point. The analysis reveals a significant negative correlation between age and daily step count, indicating that older participants tended to take fewer steps, regardless of condition. Regression analysis using Pearson correlation coefficient produced the following equation: Age=79.02−0.0077 X Daily Steps, with SE=0.0015, a coefficient of determination (*R*²) of 0.28, and a *p*<0.001.

Across all participants, Pearson correlation analysis revealed a significant negative association between age and baseline average daily steps (r = −0.53, *p*<0.001). Simple linear regression yielded the equation: Age = 79.02−0.0077 X (Daily Steps), with a slope SE=0.0015, coefficient of determination *R*² = 0.28, and *p*<0.001. These findings indicate that older participants tended to take fewer steps per day than younger individuals. These findings underscore the importance of considering age when assessing physical activity using pedometers or wearable devices, particularly after 40 years of age in men (Figure 2).

## DISCUSSION

The present study examined HE during the use of digital sham step-counting watches on ambulatory activity status in men across 3 different age groups. The results of this study revealed a significant HE combined with the use of pedometer watches on physical activity by increasing the number of steps men performed per day when wearing the device over a 7-day period. Previous studies have also reported increases in step counts through the use of tracking technologies such as watches and smartphone applications.^18^^,^^25^^,^^28^^,^^29^ However, these studies also showed some variability in step-count estimates between smartphone applications and the actual wearable device.

However, the present study showed a different increase level in men across different age groups. To date, this is the first study to precisely quantify HE in different age groups. Understanding the effect of using a pedometer on physical activity is necessary to accurately interpret the measurement of steps over a week. Such understanding can contribute to combating a sedentary lifestyle^23^^,^^28^^,^^30^ and importantly to minimizing bias in scientific research that relies on surveillance-based methods when pedometers are used to characterize participants into active and inactive.^31^, ^32^, ^33^ It also highlights the importance of developing strategies to mitigate the side effects of reactivation in behavioral trials,^34^ (see also König and colleagues^35^) or even following a hospitalization period for all ages and particularly among older individuals.

Data collected from men across different age groups in both the experimental and control groups showed a decline of daily steps as individuals age, regardless of their group. These results confirm the conclusions of numerous previous studies on the tendency towards insufficient physical activity with age.^36^^,^^37^ Indeed, it is widely recommended that individuals of all ages engage in at least 10,000 steps per day to maintain adequate levels of physical activity. Based on this guideline, the current findings indicate a consistent pattern of low physical activity among participants, classifying them as sedentary.^22^^,^^23^^,^^28^ This finding of fast decline in physical activity, especially after the age of 45 years is concerning because it is well documented that regular physical activity is essential for the prevention of various chronic diseases and constitutes a nonpharmacological alternative effective in improving the quality of life of individuals.^1^^,^^23^^,^^38^ In contrast, the greater relative increase among older adults could reflect greater responsiveness to perceived monitoring or lower baseline activity levels, resulting in a greater proportional change. The high increase in daily steps can also be due to retirement and time availability in older individuals. It is important to take feelings and interests into account when measuring physical activity levels because this study shows the effect of being monitored has a significant effect on ambulatory physical activity. Another study argues that individuals with greater interest in physical activity may be particularly prone to measurement reactivity^32^; even though the participants of the current study were contacted from the participant bank of people with interest in physical activity research, they were all sedentary and had low level of ambulatory physical activity. Moreover, being included in the participant bank can be based on an interest in knowledge from different fields such as sociology and psychology studies, which normally is performed by completing questionnaires or doing some interviews.

In addition to the step count, the speed of walking itself plays an important role in preventing morbidity and improving quality of life. The faster the walking speed (>5 km/hour), the less deterioration in quality of life is observed.^5^^,^^6^ However, not all variables related to walking were assessed in this study because of lack of sufficient resources and funding.

Men showed a significant increase in step count during the intervention week (when the watch was worn in the experimental groups), with an increase of more than 50% in all 3 age groups compared with periods without the watch. These significant increases suggest that wearing the step-count pedometers have a reactivation effect on users^39^ because of the effect of being aware of being monitored for HE. This is consistent with the results of previous studies, which show that activity trackers can encourage activity increased physicality.^10^^,^^37^^,^^40^ Therefore, the findings of this study suggest that researchers should not provide pedometers to assess ambulatory physical activity, but rather consult individuals' smartphone usage history to gain a more accurate understanding of their behavior. Furthermore, the duration of HE was related to age, awareness, and knowledge of the tool’s advantages.^41^^,^^42^ To date, studies have suggested different number of days to obtain the most reliable measurements of ambulatory physical activity.^43^^,^^44^ Therefore, the history of the performed ambulatory physical activity will remain the first suggestion of this study.

When results from physical activity screening questionnaires have been reported to suffer from social desirability bias,^45^^,^^46^ their use remains valuable for gathering information about habitual physical activity from individuals. The inclusion of objective physical measurements such as BMI, muscle mass, force, and endurance to be used in clustering analysis (different variables from the same individual) may further improve the accuracy of classification and overall assessment.^47^

However, it is also important to note that the control middle-age group showed a significant 31.84% decrease in step counts during the intervention week. This decrease could be explained by several factors, such as external circumstances (e.g., weather), that may have affected their physical activity. Additional studies would be needed to explore these dynamics in more detail.

The results of this study have an important warning for researchers when using pedometers to evaluate ambulatory activity over 1-week period. Results suggest that incorporating activity tracking devices could influence the classification of the participants’ physical activity, particularly among older men who showed the highest or significantly increased values.^10^ In addition, it would be recommended to leave the watch on for a long time until the step count per day is stable and the HE subsides over time.^48^^,^^49^ Further longitudinal studies evaluating HE over different time periods and different sex are needed.

### Limitations

Some limitations of this study should be acknowledged. First, the postintervention observation period was limited to 1 week. Longer-term studies are needed to determine whether the observed increases are maintained over time. Second, the study did not account for seasonal variations in physical activity, which could influence the results. In addition, introducing a retrospective bring-your-own-device design into the study may provide information on variability in device versions and usage patterns. Differences between wrist-based and phone-based measurements could have affected step accuracy. Moreover, the use of a mobile app could represent an additional constraint for participants, requiring them to constantly carry their phones and may underestimate true step counts when devices are not worn. The absence of demographic, socioeconomic, and health-related information further limits the generalizability of the findings.

Despite the small sample sizes per group (*n*=10), the post-hoc power analysis showed that the study was sensitive to detect the large differences observed (Cohen *d*>2.5, power>0.99). However, these estimates should be interpreted with caution, as they are based on strong assumptions of equal variance and short-term stability of measurements. Future studies with larger and more heterogeneous samples should be conducted to determine whether these results are replicable under different conditions.

## CONCLUSIONS

This study highlights the HE on ambulatory activity status when using the tracking devices showing more than 50% increase in all age groups. Also, the study reveals a strong positive reaction because of HE among users of pedometer watches and especially among the older adults. The differences between age groups highlight the importance of considering age as a factor in the assessment of physical activity status. The research group highly recommends that other researchers do not count on step-counting devices in extracting daily ambulatory activity status, especially when the device is only used for 1 week. Instead, researchers should consider longer monitoring periods to allow behavior to stabilize rather than completely neglecting step-counting measurements and combine the pedometer-derived data with the steps performance history from mobile applications.
